# Ischemic stroke rehabilitation through endurance training of varying intensity and duration in male Sprague-Dawley rats

**DOI:** 10.22038/ijbms.2025.86115.18602

**Published:** 2025

**Authors:** Moein Fasihiyan, Maryam Nourshahi, Maryam Taheri, Yasmin Asadi, Reza Pakravan

**Affiliations:** 1 Department of Biological Sciences in Sport and Health, Faculty of Sport Science and Health, Shahid Beheshti University, Tehran, Iran; 2 Department of Kinesiology and Physical Education, McGill University, Montréal, Quebec, Canada; 3 Department of Kinesiology and Recreation Management, University of Manitoba, Winnipeg, Manitoba, Canada

**Keywords:** Angiogenesis, Apoptosis, Exercise, Neurogenesis, Stroke

## Abstract

**Objective(s):**

This research aimed to investigate the effect of 2 types of exercise on apoptosis, neurogenesis, and angiogenesis factors in the penumbra area of stroke during the rehabilitation period after stroke.

**Materials and Methods:**

A transient distal middle cerebral artery occlusion (td-MCAO) model was used to induce stroke and after that, the animals were randomly divided into three groups: stroke, stroke + continuous exercise with increasing duration (CTID), and stroke + exercise with increasing intensity (CTII). At 24 hr spost-stroke , MRA, neurological deficit, and behavioral tests were conducted, and also continuous exercises were conducted for five consecutive days, Finally, MRI and behavioral tests were performed, and 24 hr after that, tissue separation and blood sampling were performed to evaluate plasma irisin, Extracellular Signal-Regulated Kinases 1 and 2 (ERK1/2) / cAMP Response Element-Binding Protein (CREB) / 90 kDa Ribosomal S6 Kinase (P90RSK) pathway, Vascular Endothelial Growth Factor (VEGF) / Vascular Endothelial Growth Factor Receptor 2 (VEGF-R2), and Brain-Derived Neurotrophic Factor (BDNF) / Tropomyosin Receptor Kinase B (TrKB) levels. for statistical analysis, one-way and two-way ANOVA tests were used at the significance level of P<0.05.

**Results:**

Both training models reduced the volume of stroke and neurological defects compared to the stroke group (*P*<0.05), while the amounts of irisin and CREB in the CTID group increased significantly compared to the CTII and stroke groups (*P*<0.01). VEGFR2 values in training groups increased significantly compared to the stroke group (*P*<0.05) but in the CTII group, VEGFR2 values increased significantly compared to the CTID group (*P*<0.05).

**Conclusion:**

The findings of the present study showed it seems that doing exercises with moderate intensities and gradually increasing the duration of exercise in the acute phase after stroke can be considered a suitable treatment in future research.

## Introduction

Physical exercises have been considered some of the most effective non-invasive therapeutic interventions aimed at improving cognitive and sensory-motor disability after stroke (1). However, research on the effect of exercise training on the recovery of stroke patients during the rehabilitation period has reported different results due to the difference in metabolic stress in various types of physical exercises (2). Some studies regarding the effectiveness of physical training on stroke have shown that higher intensity training causes better cognitive and physical performance responses during rehabilitation (3). On the other hand, it has been reported that high-intensity training increases metabolic stress and consequent oxidative stress, and performing this type of training after a stroke increases inflammatory factors and activates cell death and proteolytic pathways (4). While it has been reported moderate and low-intensity exercises after stroke augment blood circulation and activate neuroprotective pathways (5). In addition, it has been shown that one of the other important factors in performing physical exercises after a stroke is to investigate the impact of different intensities and duration trainings in the primary and secondary phases after a stroke (6, 7). Conflicting results indicate that the intensity and duration of exercises as well as the amount of metabolic and mechanical stress due to exercises after stroke play a considerable role in rehabilitation after stroke (8). Therefore, identifying and investigating the exact mechanism of various types of exercises with different intensities and duration can be considered useful to provide a suitable solution to improve the conditions of neuroprotection and other neurological factors.

As a result of exercise, the muscles release many myokines into the circulation, causing physiological changes in other organs, which are recently called Exerkines (9). Among these factors, irisin as a hormone-like myokine plays a pivotal role in the neuroprotective and upstream pathway of the neurogenesis process in the penumbra area after stroke (10, 11). Recent studies have shown that changes in irisin levels stimulate neuroprotective signaling pathways and inhibit neurodegenerative pathways in nervous system disorders (12). The signaling pathway is considered an important pathway related to apoptosis in nerve cells, which have given different responses due to different intensity physical exercises, and it has been determined that these changes play a vital role in neuroprotection (13). Moreover, Brain-derived neurotrophic factor (BDNF) plays a prime role in neurogenesis, which activates neurogenesis through the pathway in the penumbra region before and after stroke due to physical exercises (14). It also has been reported that as a result of exercise, the changes in the expression of BDNF protein and its receptor (TrKB) along with the activation of CREB improve recovery function during rehabilitation period after stroke (15). 

On the other hand, Ischemic stroke occurs when the blood vessels that supply oxygen and fuel to the nerve cells are blocked, which then causes nerve cell death in the stroke area due to acidosis and hypoxia (16), which is why the amount of blood supply to the nerve cells in the penumbra area in post-stroke rehabilitation it is very important that changes in the expression of Vascular Endothelial Growth Factor (VEGF) protein and its receptor (VEGF-R2) inhibit apoptotic catabolic factors and increase neurogenesis factors in stroke recovery (17). Previous studies have investigated the effect of physical exercises with different intensities on angiogenesis in nerve cells, which have shown that performing high-intensity interval training before stroke increases the expression of VEGF, VEGF-R2 proteins as well as reduced stroke volume compared to moderate intensity training (18).

Despite extensive research, previous studies have not explored enough how the intensity and duration of physical exercise affect functional recovery during the rehabilitation period following a stroke. Therefore, in this study, we evaluated the effects of two types of endurance training with increasing intensity and duration on the effectiveness of irisin in modulating apoptotic factors such as ERK1/2/P90RSK/CREB signaling, neurogenic and angiogenic factors such as BDNF/TRKB/CREB, and VEGF/VEGF-R2, during the post-stroke rehabilitation period in td-MCAO stroke model rats.

## Materials and Methods

### Animals and experimental groups

Thirty Male Sprague-Dawley rats (250–280 g, 8 weeks, Razi Vaccine and Serum Research Institute, Karaj, Iran) were used and manipulated in experiments according to protocols approved by the Institutional Animal Care and Use Committee (IACUC) at SBU. And also, the study was carried out by the NIH Guide for the Care and Use of Laboratory Animals and was approved by the Ethics Committee of Shahid Beheshti University (IR.SBU.REC.1401.046).

https://ethics.research.ac.ir/IR.SBU.REC.1401.046

**Figure 1:**
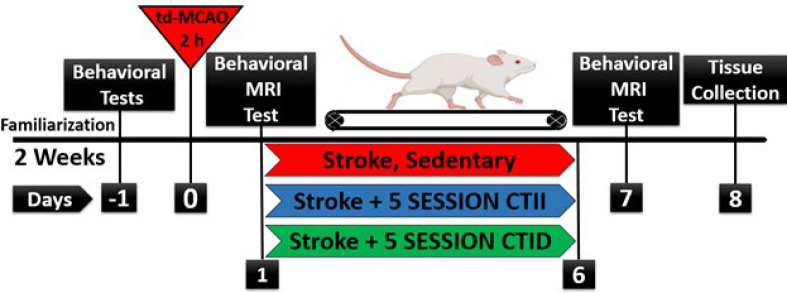
Schematic representation of the experimental design for ischemic stroke rehabilitation in male Wistar rats

Following at least one week of acclimation, animals were randomly divided into three groups: td-MCAO (N=7 (10-3)), td-MCAO + CTID (N=7 (10-3)), and td-MCAO + CTII (N=8 (10-2)) after stroke surgery in each group, rats that had different neurological deficits from the criteria (>2, 3<) of this research were excluded. Exercises started 24 hr after td-MCAO surgery in the exercise groups, while the control group had no physical activity, and to equalize the stressful conditions of the treadmill, during the exercises of the continuous exercise groups, the cage of the control group was placed next to the treadmill. Finally, animals in each group were sacrificed on day 8 after reperfusion for further analysis. Schematic study design Figure 1.

### Focal cerebral ischemia

A transient distal middle cerebral artery occlusion (td-MCAO) model was used to induce stroke in experimental groups in the present study. Rats were anesthetized using the PARKLAND inhalation anesthesia machine (made in USA) with 2–5% isoflurane inhalation anesthetic to cause temporary cerebral ischemia and were placed under a surgical microscope. After sterilizing the surface of the animal’s skin with 70% alcohol, a longitudinal incision was made in the carotid skin area, and after removing the pectoral muscles of the mammary thoracolumbar, the middle carotid artery was identified in the anterior neck area, and after separating this vessel from the vagus nerve, the artery was occluded by a special clamp, and then a skin incision was made between the left eye and the ear, and the temporal muscle was held aside and removed by a small skull bone drill in the temporal section, followed by the middle cerebral artery (MCA) after craniotomy. It was exposed and using a 9-0 nylon thread, the distal part of the left MCA was ligated at the bifurcation site into the frontal and posterior branches. After two hours, the suture knots were gently cut with microscissors to allow blood flow again in the ischemic areas of the brain. Then the wound was sutured and the animals were kept in the animal recovery box at a temperature of 37 °C until complete recovery from anesthesia (19).

### Inclusion criteria and double-blind measurement

Neurological evaluations were performed by an experimenter without knowing the type of intervention, experimental groups, surgeries, and treatments. Animal cages as well as tissue samples were coded before allocation for analysis.

### Neurological deficit

After t-dMCAO surgery, the neurological deficit scores (NDS) were calculated in rats after 24 hr (20). The scoring method was: grade zero, absence of neurological deficit. Grade 1, inability to fully extend the forelimb. Grade 2, when the animal is pulled by the tail, it turns around more than normal. Grade 3, unilateral rotation around itself. Grade 4, lack of spontaneous movement and low level of consciousness. And grade 5, death. Rats with a score of 0, 4, or 5 were excluded from the study. In this study, according to the type of t-dMCAO stroke intervention, animals with a neurological deficit score of 2 and 3 were included in the experiment.

### Exercise protocol

The desired exercise protocol was generally performed on a treadmill for 5 sessions in a week. These exercises were designed based on the research background in the field of post-stroke rehabilitation, according to the 2nd and 3rd grades of the NDS test. In the first session (24 hr after stroke surgery), the animals performed a warm-up activity at a speed of 5 m/min for five minutes, and then a continuous activity was performed for ten minutes at a speed of 10 m/min. And finally, cool-down was done at a speed of 5 m/min for five minutes. In the group of endurance training with increasing duration (CTID), the duration of each session was increased by two minutes compared to the previous session, which was ten minutes in the first session and 18 min in the last session, and in the group of endurance training with increasing intensity (CTII) the speed of the treadmill increased by 2 meters per minute in each session and reached 18 meters per minute on the last day. According to the exercise volume component, both groups were equal in volume based on the distance (Table 1).

### MRI

Stroke volume was estimated 24 hr and 7 days after the intervention using a 3.0 T MRI scanner (MagnetomVerio, SIEMENS, Germany). T2-weighted imaging (T2WI) was conducted to detect the infarct volume from brahma +1.7 to −6.3 mm using a T2-SPACE sequence with the following parameters: TR = 1,000 ms, TE = 155 ms, FA = 120◦, FOV = 60 × 60 mm^2^, matrix = 192 × 192, and 72 slices of 0.4 mm thickness without a gap (21).

### Western blot

Western blot was performed, according to a previously described method (Kim *et al.*, 2015). Protein extracts from cortical areas around the infarct tissues (30 μg) were separated by sodium dodecyl sulfate-polyacrylamide gel electrophoresis. Electrophoresis (10% SDS-PAGE gel) was performed using 10% polyacrylamide with 0.05% bis-acrylamide. Gel transfer to a PVDF membrane was performed under 200 V for one hour, and the blots were probed with anti-CREB rabbit polyclonal antibody (1:500, Abcam, MA, USA), anti-ERK1/2 rabbit polyclonal antibody (1:1,000, Santa Cruz Biotechnology), rabbit anti-phospho-p90RSK (p-p90RSK; 1/1000, Cell Signaling, USA), anti-BDNF monoclonal antibody (1:1000; Abcam, MA, USA), anti-TrKB rabbit polyclonal antibody (1:1,000, Santa Cruz Biotechnology), anti-VEGF mouse monoclonal antibody (1:1,000, Santa Cruz Biotechnology), anti-VEGF Receptor 2 Rabbit polyclonal (1:1000; Abcam, MA, USA), and anti-rabbit IgG-HRP by ECL Western Blotting (1:1000; Santa Cruz Biotechnology) were used as the secondary antibodies. The blot images for each antibody were analyzed using an image analysis program (ImageJ 1.52.V. National Institutes of Health, Bethesda, MD, USA) to measure protein expression based on relative image density.

### Elisa

The irisin levels in serum were measured by a commercial ELISA kit (Irisin recombinant Elisa Protocol, EK-067-29, Phoenix Pharmaceuticals, Schiltigheim, France). The limit of sensitivity was fixed at 1.29 ng/m. Directions were performed according to the manufacturer’s instructions, with samples diluted at 1/10. All assays were performed in duplicate, and a positive control validated the experimental conditions.

### Behavioral tests

*Rotarod*

To assess the motor behavior recovery after td-MCAO in different groups, rats were subjected to a Rotarod test. In this experiment, the rats were placed in a rotating rod with protocol from 5 to 40× rpm/min during the test period, and finally the latency to fall off the rotating rod was recorded three times in 24 hr and day 7 after td-MCAO surgery (22).

### Grip strength

Forelimb grip strength performance test was performed on the 1st, 3rd, and 7th days after stroke in the right and left forelimbs separately by (animal dynamometer, DSA, Iran).

### Adhesive removal

In order to evaluate the function of cerebral hemispheres, the adhesive removal test was performed at baseline, days 1, 3, and 7 post-stroke. Small adhesive papers (The size of the palm of the forelimb) were simultaneously applied, and the latency to contact and removal of the adhesive paper on the right and left side was recorded in four trials with a maximum removal latency of 180 sec per trial.

### Statistical analysis

Statistical analyses were performed using SPSS Statistics for windows, Version 21.0 (SPSS Inc., Chicago, IL, USA). the normality and homogeneity of data were analyzed using Shapiro–Wilk and Levene’s test, respectively. One-way analysis of variance (ANOVA) followed by Tukey’s *post hoc* test was used to determine irisin and protein levels. Moreover, repeated measures of ANOVA followed by Bonferroni *post hoc* test were used to analyze behavioral tests. Values are expressed as mean±SD, and the probability level of statistical significance was set at *P*<0.05.

## Results

### MRI measurement, weight, and neurological deficit

MRA imaging was done before and after a week of endurance training, and after that densitometry was done using Image J software (ImageJ 1.52.V. National Institutes of Health, Bethesda, MD, USA), which showed significant reduction of stroke volume in the CTID group (*P*<0.05), Figure 1 A,B. Weight changes were measured on the 1st, 3rd, 5th, and 7th days, after the stroke, a significant weight loss was observed in all groups (*P*<0.05) and finally, the CTID and CTII training groups had more weight gain than the control group; however, significant changes were observed only in the CTID group (*P*<0.05), Figure 1C. A 5-point system assessed neurological deficit at 24 hr and 7 days after the stroke, and the results showed that exercise training reduced neurological impairment compared to the stroke group (*P*=0.0189) and these changes were greater in the exercise group with increasing duration (*P*<0.05), Figure 1D.

### IRISIN and expression of apoptotic proteins

**Figure 2:**
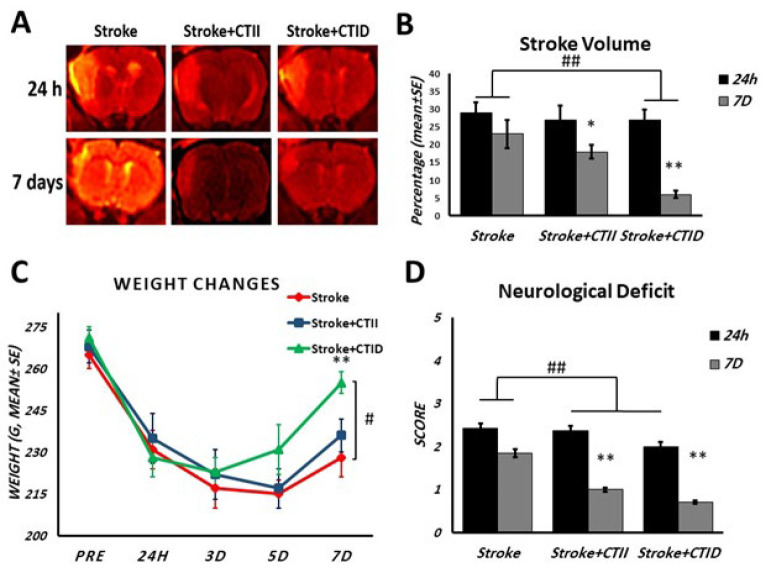
(A) MRI images with Hot Iron mode, 24 hr and 7 days after stroke and performing CTID and CTII in different groups of male Sprague-Dawley rats. (B) Quantification of the ischemic area based on the percentage of the infarcted area relative to the total brain volume at 24 hr and 7 days after stroke in male Sprague-Dawley rats. (C) Weight changes due to pathological factors on different days, and (D) Neurological deficit test score in male Sprague-Dawley rats

Plasma Irisin levels in the CTID group increased significantly compared to other groups (*P*<0.001), while no significant difference was observed between the plasma Irisin levels in the CTII and Stroke groups (*P*>0.05), Figure 2A. CREB protein levels in the penumbra region increased in both endurance training groups compared to the stroke group, and these changes were significant only in the CTID group (*P*<0.05), Figure 2B. However, the ERK1/2 protein levels were higher in the training groups than in the stroke group, but these changes were not statistically significant (*P*>0.05). Additionally, the values of P-P90RSK protein in the CTID and CTII groups were significantly increased compared to the stroke group (*P*<0.01 and *P*<0.05, respectively), Figure 2 C, D.

### Expression of BDNF, TrKB, VEGF, and VEGF-R2

**Figure 3:**
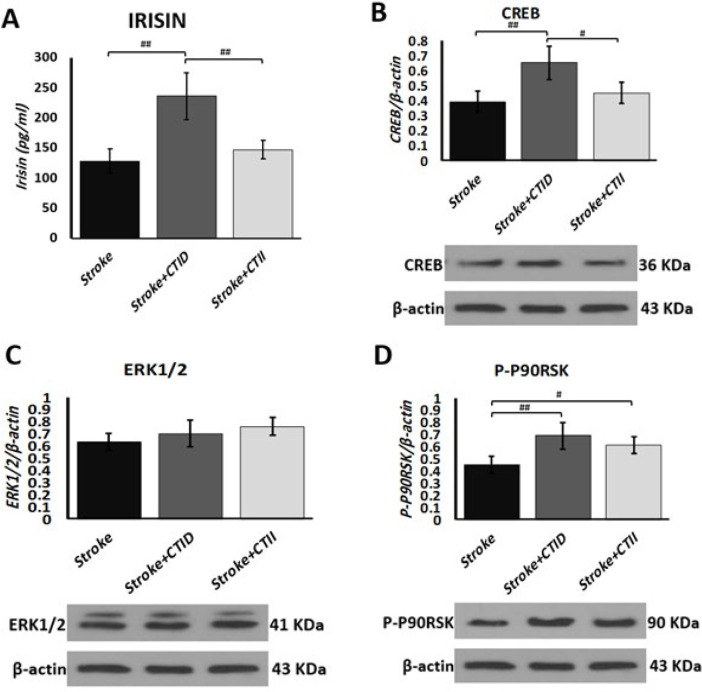
(A) Plasma irisin levels 7 days after stroke and endurance training (CTID and CTII) in male Sprague-Dawley rats. (B–D) Expression of apoptotic-related proteins (CREB, ERK1/2, and P-P90RSK) 7 days post-stroke; significant group differences identified via one-way ANOVA (# *P*<0.05, ## *P*<0.01).

Continuous training with increasing duration in the acute phase after stroke significantly boosted the levels of neurogenic proteins BDNF in comparison with other groups (# *P*<0.05, Figure 3A), its receptor TrKB compared to CTII (#*P*<0.05), and stroke (##<0.01, Figure 3B). Also, VEGF protein values increased significantly in the CTID group compared to the CTII and stroke groups (# *P*<0.05, Figure 3C), although no significant difference was observed between the CTII and stroke groups (*P*>0.05). In spite of the fact that the values of VEGF receptor 2 (VEGF-R2) increased in both training groups, the significant changes of VEGF-R2 in the CTII group were more compared to the CTID group (#*P*<0.05) and compared to the stroke group (##*P*<0.01) and Also, a significant difference was observed between the CTID and stroke groups (#*P*<0.05, Figure 3D).

### Behavioral and functional assessments

**Figure 4:**
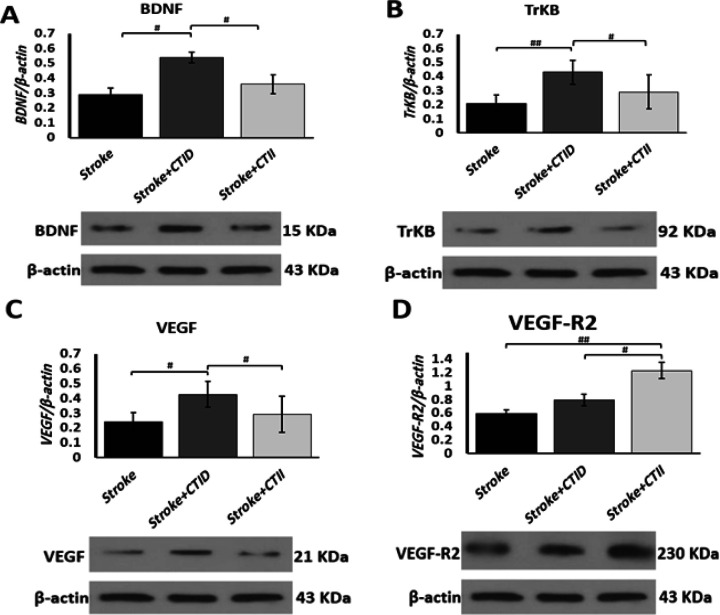
Continuous training at varying intensities and durations significantly altered neurogenic and angiogenic markers during stroke rehabilitation in male Sprague-Dawley rats

As shown in Figure 4A, the duration of resistance to fall in the Rotarod test 24 hr after stroke significantly decreased in all groups, and then on days 3 and 7, the time of latency to fall in CTID group had significant increase in comparison with CTII AND stroke group (*P*<0.05, *P*<0.01). The strength of the front limb in the Grip Strength test also increased significantly in training groups compared to the stroke group after 3 and 5 training sessions (*P*<0.05). while between CTID and CTII there were no significant change (P>0.05) Figure 4B. Also, in the Adhesive Removal test (Figure 4C), the time to touch and time to remove in the training groups was significantly decreased compared to the stroke group after 3 and 7 days (*P*<0.01). But between continuous training groups CTID group had significant decrease compare with CTII (*P*<0.05). 

## Discussion

The results of this study approved the effects of endurance training with different intensities and durations on reducing the apoptotic protein functions and raising neuroplasticity and angiogenesis factors in the cortical and peri-ischemic region and also functional outcomes obtained during the rehabilitation period after ischemic stroke. Specifically, our results showed that regardless of the intensity and duration of training, in training groups stroke volume declined and the weight of animals increased after seven days. While the amount of irisin was boosted by 85% in just the training group with increasing duration (CTID).

The reduction of stroke volume and changes in neurotrophic factors aligned with, a study that investigated the effects of different exercise intensities after ischemic stroke which stroke volume was decreased after short time of training sessions and also reported that the levels of BDNF, TrKB, and NGF in the training groups increased significantly compared to the control group during 3 and 14 days after stroke (5). Another study has shown that starting exercise 24 hr after stroke can reduce stroke volume and apoptotic factors such as caspase 3 and 8 as well as increase the main angiogenesis marker (VEGF) in the stroke area on days 3 and 5 after stroke (6). In addition, has been determined that performing continuous exercises 24 hr after a stroke at a speed of 12 m/min for 30 min, 5 days/week for 4 weeks, caused a significant reduction in stroke volume and improved neurological function (21). In this regard, the effect of different intensities of exercise after a stroke has been investigated, the high-intensity endurance training protocol includes running on a treadmill at a speed of 30 m/min for 30 min and moderate-intensity endurance exercise, ten minutes at a speed of 5 m/min, ten minutes 9 m/min, and ten minutes 12 m/min, which the results showed performing endurance exercises significantly reduced the infarct volume and increased neuroplasticity and angiogenesis markers including HIF-1α, BDNF, TrkB, and CREB (14).

While in the present study, the possibility of performing exercises was not associated with the intensity and duration of the exercises in the previous studies (5, 6, 14), and the high intensity of the exercises was not applicable for the animals after stroke, on the other hand, a study was conducted to investigate the effect of exercises after stroke on neurological factors, physical exercise was used at an intensity of 2 to 8 meters per minute for ten minutes, and the exercises started 24 hr after the stroke and were performed for 6 consecutive days, and the results showed that performing physical exercises improves inflammatory factors and neurological condition after stroke (23). In addition, has been investigated the effect of exercise intensity 24 hr after stroke in seven days, and the results of this study showed that the highest amount of BDNF was observed as a result of low-intensity exercises and gradually increased intensity exercises after stroke, there was a significant increase in BDNF compared to the high-intensity exercise group, which the protocols included low-intensity exercise group at a speed of (5 m/min, 30 min), the high-intensity exercise group (26 m/min, 30 min), and gradually increased intensity group (5–26 m/min,30 min) for 7 days after stroke (24). While, in our research, the intensity and duration of exercises were designed and performed incrementally, and the results showed that performing exercises with constant intensity and increasing duration caused better effectiveness than the group of exercises with increasing intensity in reducing stroke volume and neurological disorders.

Not only in previous studies, different exercise intensities have been investigated but also, the time of starting rehabilitation exercises after stroke seems to be very important as well. The research examined the effect of starting physical exercises at 6, 24, and 72 hr after a stroke. The results showed that starting physical exercises within 6 hr after the stroke increased the apoptotic factors such as caspase 3 and Bax/BCL-2 ratio while 24 and 72-hour groups showed improvement in these apoptotic factors (25). Tani *et al.* have illustrated the commence of endurance training 3 days after the stroke, with the intensity of (8–15 m/min, 20 min/day, 5 days/week) for 4 weeks, the amount of BDNF, NGF and TRKB proteins increased in the exercise group compared to the ischemic reperfusion group (26). In this study, exercises were started 24 hr after the stroke, and 5 sessions were performed in one week, and the results showed that BDNF and tyrosine kinase B values were 71.5% and 83%, respectively increased in the CTID group compared to the stroke group.

Irisin has recently been noticed as one of the main factors influencing neurogenesis pathways such as BDNF and ERK1/2/AKT after stroke (27, 28). The results of our research showed that plasma irisin levels in the CTID group increased by 85% compared to the stroke group and 61% compared to the CTID group.

The results regarding the changes of irisin values due to training with different intensities have many contradictions. Contrary to the results of the present study, Yani *et al.* recently reported that performing high-intensity exercises caused a significant increase in irisin and BDNF levels after stroke, while moderate-intensity exercises did not significantly increase irisin levels (29), it was found that plasma irisin concentration and intramuscular FNDC5 protein gene expression decreased after stroke, and it was reported that there was an inverse relationship between irisin levels and stroke volume, neurological deficits, and TNFα and interleukin-6,1β concentrations, on the other hand, Irisin treatment increased the expression of ERK1/2/AKT proteins, and in the training group that used irisin inhibitor, more severe neurological damage was observed than in the control group (30). It has been reported that moderate-intensity aerobic exercise inhibits neurodegenerative factors like NF-κB/NLRP3/5-HT in post-stroke depression by increasing irisin expression (31). According to the results of the present research, Leger *et al.* showed that moderate-intensity aerobic exercise was associated with a greater increase in irisin and BDNF levels in the Rats hippocampus (32). Also, human studies showed that 8 weeks of physical training increased irisin in the blood circulation of people after stroke (10).

Angiogenic factors in the present research in the continuous training group had a significant increase compared to the control group, and the values of VEGF in the CTID group increased by 49% compared to the CTII group and 52.7% compared to the stroke group, while the values VEGF-R2 protein increased by 56% in the CTII group compared to the CTID and 109% compared to the stroke group. a research investigated the effect of high-intensity interval training after stroke, and the results showed that high-intensity interval training significantly increased VEGF (33). Geng *et al.* reported that continuous physical exercise 24 hr after stroke with an intensity of 15 m/min and a duration of 30 min increased VEGF levels in the Ischemic Penumbra region on days 3 and 5 after stroke and physical exercise (6). Regarding the regulatory pathway of ERK extracellular kinase1/2 and CREB in relation to neuroprotective effects, the amounts of proteins related to the neuroprotective pathway ERK1/2 were not significantly different between the groups, but CREB values in the CTID group increased by 67% compared to the stroke group and 48.9% compared to the CTII group. In this regard, Li *et al.* investigated the effect of high and low-intensity exercise training after stroke induction in 3, 14, and 28 days, in which protein factors such as tau, GAP-43, PSD- 95 evaluated SYN as synaptic proteins and HI1α, BDNF, NGF, TRKB, and CREB proteins as neurogenic proteins in the hippocampus. The results showed that the intensity of the exercises will be an an effective factor in the expression of proteins related to rehabilitation in the synapse and hippocampus sections, in the meantime, the results indicated that moderate-intensity exercises in 3, 14, and 28 days after stroke, they had more effects than high-intensity exercises, and in addition, factors such as stroke volume and improvement of behavioral performance in the moderate-intensity exercise group had relative superiority compared to high-intensity exercise (5). Lee *et al.* reported in a study that as a result of ischemic stroke, the ERK-Akt-CREB-BDNF signaling pathway is severely inhibited in the hippocampus and worsens short-term memory through the activation of apoptotic pathways. In this regard, they investigated the effect of low-intensity continuous exercise that was performed for 14 days after a stroke. The training protocol consisted of 30 min of sports activities, which were performed at speeds of 3, 2, and 5 meters per minute, each for ten minutes, continuously for 14 consecutive days. The results of this research showed that low-intensity continuous training increases the phosphorylated ratio of cyclic adenosine monophosphate binding protein (P-CREB/CREB) and protein kinase B (P-AKT/AKT) and also increases BDNF and TRKB protein levels in the hippocampus of stroke model rats became ischemic (34).

The timeline includes stroke induction, endurance training (CTID and CTII), and assessments at 24 hr and 7 days post-stroke

The results of the ANOVA tests are marked with significant signs (* *P*<0.05, ** *P*<0.01) as within-group changes and (# *P*<0.05, ## *P*<0.01) between groups

**Table 1:** Continuous training protocol with increasing intensity and duration in male Sprague-Dawley rats

**Total distance** **meter**	**Day 5** **m/min × min**	**Day 4** **m/min × min**	**Day 3** **m/min × min**	**Day 2** **m/min × min**	**Day 1** **m/min × min**	
700	10×18	10×16	10×14	10×12	10×10	CTID
700	18×10	16×10	14×10	12×10	10×10	CTII

CREB: cAMP response element-binding protein; ERK1/2: Extracellular signal-regulated kinases 1/2; P-P90RSK: Phosphorylated p90 ribosomal S6 kinase

(A) BDNF levels increased in the CTID group. (B) TrkB receptor increased with training intensity and duration. (C, D) VEGF and VEGF-R2 were elevated in CTID and CTII groups. Significance: *P<0.05, **P<0.01 (within-group); #*P*<0.05, ##*P*<0.01 (between groups).

BDNF: Brain-derived neurotrophic factor; TrkB: Tropomyosin receptor kinase B; VEGF: Vascular endothelial growth factor; VEGF-R2: Vascular endothelial growth factor receptor 2

**Figure 5:**
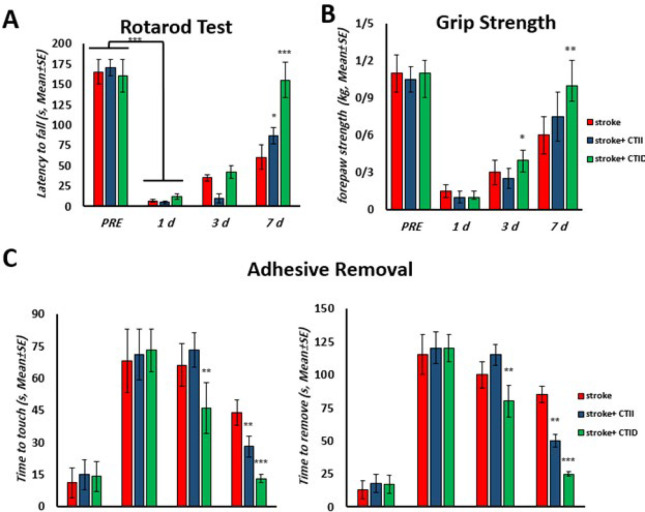
Stroke induction and physical exercise caused cognitive-motor changes in male Sprague-Dawley rats over time

(A) Rotarod test showed reduced balance duration 24 hr post-stroke in all groups, with significant improvement by day 7 in CTID and CTII groups. (B) Grip strength increased significantly in the CTID group on days 3 and 7; CTII showed a smaller increase on day 7. (C) Adhesive removal test times decreased significantly in CTID and CTII groups on days 3 and 7, indicating improved sensorimotor function. CTID: Continuous exercise with increasing duration. Significance: *P<0.05, **P<0.01, ***P<0.001.

## Conclusion

Finally, the results of this research showed that exercises with increasing duration for improving neurological disorders and motor-cognitive performance are more effective than high-intensity exercises, and physiologically, physical exercises can have different effectiveness due to metabolic, inflammatory, and mechanical pressure. in the improvement of neurological problems after stroke, by examining these factors, the protocols used in this research can be used in future research to examine the path of irisin’s effect on apoptotic, neurogenic, and angiogenic factors in the acute and chronic phase after stroke.

## Declaration

The authors confirm that this manuscript is original, has not been published previously, and is not under consideration for publication elsewhere. All authors have reviewed and approved the final version of the manuscript. Ethical approval for animal experiments was obtained from the appropriate institutional ethics committee. We also acknowledge the use of ChatGPT (OpenAI, 2025) for grammar checking and improving the fluency of the manuscript.
